# Discriminatory practices and poor job performance: A study of person-related hostility among nursing staff

**DOI:** 10.1016/j.heliyon.2023.e14351

**Published:** 2023-03-07

**Authors:** Nadia Noor, Saqib Rehman, Yasmeen Ahmed, Muhammad Sarmad, Rashid Mehmood

**Affiliations:** a Department of Management Sciences, Lahore College for Women University, Pakistan; b Department of Architecture, Lahore College for Women University, Pakistan; c Riphah School of Leadership, Riphah International University, Islamabad, Pakistan; d Division of Management and Administrative Science, UE Business School, University of Education, Lahore, Pakistan

**Keywords:** Horizontal hostility, Person-related hostility, Gender discrimination, Lack of administrative support, Poor job performance

## Abstract

This study highlights the organisational-level factors that become the reason for propagating hostile behaviours among female nurses. Freire’s theory of oppression has been used as an underpinning theory for developing the conceptual framework. This study empirically verifies the conceptual framework of the study that gender discrimination and lack of administrative support are the antecedents of person-related hostility, which is the reason for poor job performance in the healthcare sector. We applied a quantitative research approach, using questionnaires to collect data. Total of 707 survey forms were collected from female nurses working in public sector hospitals in four main cities of Pakistan. The software SPSS 20 and SmartPLS 3 were used for the final data analysis. All hypotheses regarding the direct and indirect relationship of variables were accepted. Gender discrimination and lack of administrative support were positively associated with person-related hostility. Moreover, person-related hostility also mediated the relationship between independent variables (gender discrimination, lack of administrative support) and dependent variable (poor job performance). Future research is directed to study person-related hostility among nursing staff of semi-urban areas and small towns with low literacy rates, considering other dependent variables like burnout, mental well-being, and mental health. HR strategies and policies for fair performance evaluation and timely promotions of nursing professionals are proposed in the study for building an overall healthy environment.

## 1 Introduction

Many factors affect human beings, which can be investigated and analyzed better socially rather than medically. A significant factor that requires thoughtful consideration in contrast to other facets of health is the social facet of health [1]. This facet replicates the experience of individuals in the social environment and directs how they resolve social challenges [2]. The concept of horizontal hostility is one of the different dynamics that affect the workplace environment internally and result in counterproductive work behaviours [[3], [4], [5]]. Horizontal hostility is a type of workplace violence among females [6]. In different forms of horizontal hostility, person-related hostility is dangerous. It is the distressing behaviour of one worker towards another having the same rank within a chain of command that tries to find ways to influence and control the individual by disrespecting and waning her status [7,8]. Person-related hostility is a type of horizontal hostility which has never been explored from the antecedents and outcomes point of view.

Kingma [9] included race, gender, religion, social position, lifestyle, disability, political convictions, and geographical nationality in discrimination. Discrimination describes a person or group’s deprivation and impairment due to inclusion in a particular social class [10]. In social sciences research [11,12], the literature indicates that gender discrimination or inequality prevails in different jobs. In the UK, male nurses are over-represented. They have more opportunities for higher pay bands after qualifying as compared to females [13], whereas in the USA, male nurses were paid 27.9% higher than female nurses [14]. Even as gender discrimination in healthcare is a complex issue, explanations mainly focus on organisational systems and practices [15]. Moreover, organisations must identify any behaviour related to horizontal violence, bullying, isolation, and partiality [16,17]. McCormack et al. [18] argued that behaviours related to hostility perhaps not be misunderstood or dispirited by organisational leaders and managers. Institutional norms may promote person-related hostility or institutional stress that positively correlates with bullying behaviours and horizontal hostility [19]. Therefore, support is required at all administrative levels and from nursing leaders in healthcare settings. Nursing schools must also understand and eradicate person-related hostility to ensure patients' safety by establishing a healthy work environment [20].

Longo and Newman [21] described person-related hostility as hostile behaviour propagated by one colleague towards another, either implicitly or explicitly. This hostile behaviour can be verbal, physical, or emotional. Person-related hostility refutes another’s fundamental human rights and indicates an absence of respectful behaviour and appreciation for others' value and success [22]. Bloom [23] defined person-related hostility among nurses as violence directed towards their peers through words, actions, and behaviours. Such negative behaviours control, degrade, humiliate or injure the dignity of others. These hostile behaviours negatively impact psychological health, and nurses experience communication barriers, increased stress and concentration difficulties [24]. Person-related hostility is a noticeable multifaceted and multicultural problem resulting in substantial psychological outcomes for nursing recipients and patients in the healthcare sector. Moreover, person-related hostility in healthcare not only affects nursing professionals and their work, but these negative behaviours potentially affect staffing which is a significant concern [25]. The era needs to recognise and eliminate bullying behaviours and horizontal hostility from all administrative levels through leadership to ascertain a healthy and peaceful workplace where safety and quality patient care are witnessed [20]. For this, a comprehensive research study has become necessary to identify the organisational causes of hostile behaviours to create an environment conducive to patient safety and quality healthcare.

The primary purpose of conducting this study is to highlight critical factors in the form of antecedents that cause female workers to turn into hostile behaviour toward other female peers and the expected outcome of that particular behaviour. This study is vital and substantially contributes to human resource management and psychology, specifically in the health sector. Firstly, person-related hostility among females is dangerous for personal and organisational performance; therefore, timely diagnosis and exterminating the antecedents of hostile behaviour is necessary to enhance their job performance. Secondly, the concept of male supremacy in dominant male organisations should be controlled. This study will be an eye-catching factor for policymakers to identify the presence of gender discrimination as it may badly damage organisational performance. Thirdly, due to gender discrimination, female employees are exposed to stress in their career advancements in biased societies, which urge them to become violent against the same gender or to have poor job performance. Therefore, organisations must understand that a lack of administrative support may worsen the situation. Lastly, this study has highlighted gender discrimination and the lack of administrative support in organisations for generalizability but explicitly focused on the nursing profession to main the specificity of research.

The problem is that female nurses in Pakistan face hostility and mistreatment in healthcare work settings. Many times, it is women who inflict psychological hatred and hostility against each other. So the need is to investigate the causes and adverse outcomes impacting women’s work performance. This research study intends to answer the following research questions.

1. What is the impact of organisational factors (gender discrimination and lack of administrative support) on the incidence of person-related hostility and its outcome (poor job performance) among female nurses working in the healthcare sector of Pakistan?
2. What is the mediating impact of person-related hostility on the relationship between organisation factors (gender discrimination and lack of administrative support) and its outcome (poor job performance)?

The empirical literature has been cited for developing propositions based on the above research questions. For data analysis, the reflective measurement and structural model are applied to evaluate reliability, validity, and hypothesis testing. Research findings have been discussed with strong arguments from the empirical literature. In the conclusion section, research implications, limitations, future directions and policy recommendations are stated to highlight the contribution of this study.

## 2 Review of literature

Freire’s model of oppression [26] describes oppression’s results in the act of violence; therefore, the violence of the oppressors results in the violence of the oppressed in reaction. The oppressors view resistance or punitive violence of the oppressed as criminal behaviour and try to keep the peace by forcing the oppressors to be downcast. As soon as the oppressed achieve equal status and equality of expression in their lives, then previous oppressors feel oppressed. They have practised different ways to oppress others, and when their domination and power are taken away, they think their dominance and authority manipulation have been grabbed. They become narcissistic and self-centred and seem unable to consider every person worthwhile of equality and justice treatment. Powerlessness and inability of the oppressed contribute to this negative behaviour because they would be cruelly penalised if they reacted to the dominant individuals who control their lives. Nowadays, the term horizontal hostility describes how women target other women who seem to be prominent due to professional success [27]. Horizontal hostility explains power as domination amongst women. Therefore, it should be discussed in the context of power relations among women. The feminist movement for identification and provocation of male dominance pronounces that men only oppress women. Still, they negate women’s behaviour towards other women. It can never be desperate assuming that women are habitually exempted from male racist norms, attitudes and actions. Male domination should be kept in mind as the primary opponent. On the other hand, oppression is institutionalised for women, and due to the existing state of affairs, it is very easy for them to assume inconsiderately the behaviours that strengthen forms of domination [27].

Therefore, Freire’s theory of oppression has been adopted as the underpinning theory for the development of the conceptual framework of this study, and relevant support from the literature will be discussed in support of hypotheses and framing of a conceptual framework.

### 2.1 Relationship between gender discrimination, person-related hostility and poor job performance

In literature, gender discrimination has been described in two forms, i.e., sticky floors and glass ceilings. Chi and Li [28] stated that a sticky floor arises from comparing male and female employees at the same scale or rank, but males are appointed or given importance comparatively further up the scale. Shambaugh [29] described the glass ceiling as an unseen obstacle prohibiting females from advancing to high-ranking positions within an organisation, even if they carried significant achievements or credentials. So, gender discrimination in the workplace may occur because of the consequences of the glass ceiling and sticky floors, which turn the behaviours into person-related hostility. There is a school of thought which believes that institutional barriers created by men in the form of gender discrimination hinder women’s professional success [30,31]. At an organisational level, sticky floors and glass ceilings have been identified as dimensions of gender discrimination. They are used for relative hazards related to females' professional opportunities when moving up the professional ladder; it alludes to the growing difficulties for women [29]. One of the topmost objectives of women’s drive in society is to struggle for impartiality with men, as women have to compete directly with men within the corporate sector [32]. However, when several women struggled for their rights against men and attempted to attain equal status within male-dominated institutions, other women discouraged female coworkers through gossip and sabotage of progress [33,34]. These twofold and competing tactics of women to attain top-level management positions resulted in their failure to understand that they may be competing fiercely with one another compared to men. Consequently, competition among females has substantial implications for their advancement in professional careers.

Kennedy [35] defined hostility as the perception of the individuals of the same oppressed group (females) who fight against each other rather than powers of disparity that are oppressing them (male-dominated structures). In the workplace, gender schemas exist, and implementing them may result in hostility among professional females creating double-bind situations for them [36]. Professional females may become oppressed and remain one step behind men. These professional females may challenge existing conditions and threats being ignored by other female colleagues [37]. Therefore a direct relationship may be observed between gender discrimination in the workplace and person-related hostility. Based on the above-discussed literature, we can hypothesise the following:

H1a Gender discrimination is positively related to person-related hostility.

Even though the glass ceiling may describe females' lack of career progression to higher-level management positions, it is also essential to identify females' professional sufferings and hindrances because of other females [38]. Women may compete more aggressively with others for limited high-rank management positions than men [39]. Therefore, horizontal hostility provides the theoretical basis for such investigation. Therefore, male-dominated repercussions of horizontal violence, as a consequence of the glass ceiling, advocate that women’s lack of progression to high-level jobs may be due to competition among aspiring women as the number of top-level management ranks for them is limited [40,41]. The frustration of transgressing boundaries results in horizontal hostility. It is easier to fight with peers horizontally than to fight the oppressors vertically, and sometimes to fight herself in the form of poor job performance is more accessible. The oppressed or marginalised group members learn the dominant values of oppressors, who, in line, victimise each other and ultimately show less inclination towards work and poor job performance as resultant [42,43]. Therefore, based on the above-discussed literature, we can hypothesise the following:

H1b Person-related hostility mediates the relationship between gender discrimination and poor job performance.

### 2.2 Relationship between lack of administrative support, person-related hostility, and poor job performance

Freire [26] states that the people who oppress others, degrade themselves in reality and provoke the procedure that keeps them unaware of how their power, dominance, and cunning behaviour are self-destructive. The researcher pinpointed horizontal hostility as the negative behaviour of the oppressed when they target their relations; the oppressor is present amongst them, and they strike against him indirectly, consistent with an additional feature of behaviour that prevents change [44,45]. Lack of administrative support is a significant reason for dejection among the oppressed [46,47]. The oppressed people seem emotionally helpless, and earlier, they realise their reliance, expressing their frustration and desperation at times by drinking at home; it may be the only way of exit for them [48,49].

The feelings of uncertainty due to lack of administrative support provoke subordinates to inflict horizontal hostility to express themselves more powerfully [50,51]. Heim et al. [52] described that other women might get offended and annoyed when an aspiring woman achieves career success and enhances her influence, self-confidence, and power. They approach other women for help and support to emasculate competitors' success through covert aggression such as gossip. The subordinate female expresses jealousy and hurt due to the non-availability of administrative support, low confidence, and self-respect [49,53]. Moreover, subordinates' feelings of subservience and inequality result in direct aggression or person-related hostility. Therefore, based on the above-discussed literature, we can hypothesise the following:

H2a Lack of administrative support is positively related to person-related hostility.

Most women perceive other women’s success and career development negatively, and instead of supporting them, they try to discourage them. This discrete and covert behaviour describes another feature of horizontal violence expressed by women [54]. Competent and talented women do not want to appear superior due to fear of social ostracising and keep themselves from aspiring to excel. Women discourage aspiring women and like everybody at the same hierarchy level [55]. They quickly accept the most unpleasant about each other and will try to control aspiring women through gossip and further indirect aggression. Heim et al. [52] described that because of sabotage, work relationships between females lean towards conflict more than their relationship with males. This negative behaviour results in a less friendly and distressing work environment. It diminishes the prospects for collaborative and compassionate work teams within women’s workplaces, which also affect their job performances. Therefore, based on the above-discussed literature, we can hypothesise the following:

H2b Person-related hostility mediates the relationship between lack of administrative support and poor job performance.

### 2.3 Relationship between person-related hostility and poor job performance

Though, empirical literature specifically related to the strong causal or correlational effects of horizontal hostility on patient safety and quality of patient care is at the beginning stage [55,56]. Nurses' responses to horizontal hostility result from unpleasant situations wherein they are ridiculed for their contributions, their intelligence and professional abilities are threatened, and their self-esteem is severely eroded [46]. Female recipients of hostility try to avoid interfacing with colleagues who target them through abuse and insulting behaviours [[57], [58], [59]]. This hostile behaviour is not confined to the deteriorated relationship between the two groups. It also affects individuals who are witnesses, and teamwork is sabotaged in the form of poor job performance [60,61]. Horizontal hostility also contributes to the integrated factors of poor job performance, like professional disengagement, job dissatisfaction, and increased turnover intentions among nursing professionals [62,63]. Shahzad and Malik [64] conducted a research study to highlight the issue of workplace violence toward nursing professionals in the healthcare sector of Pakistan. They have stressed that this area is unexplored due to the scarcity of data. The study’s findings revealed the confirmation of nurses about experiencing violence and verbal abuse, but that remained unreported, as they believed that it is useless to report management because there would be no action to control these behaviours. Most nurses described their experience of anxiety and stress, lower job satisfaction and performance levels, high turnover intentions, and absenteeism due to workplace violence.

Hauge et al. and Aziz et al. [65,66] argued that enhanced mindfulness of prevailing problems because of an unsafe work environment results in occupational stress and psychological, physical, and organisational outcomes but also significantly impacts nurses' overall job performance. Tee, Ozcetin, and Russell‐Westhead [25] described person-related hostility as a noticeable multifaceted and multicultural problem resulting in substantial psychological outcomes for nursing recipients and patients in the healthcare sector. Individuals who experience bullying and horizontal violence have low self-respect and poor job enactment that directly impact patient safety and care [67,68]. Therefore, based on the above-discussed literature, we can hypothesise the following:

H3 Person-related hostility is positively related to poor job performance.

### 2.4 Conceptual framework

**Fig. 1:**
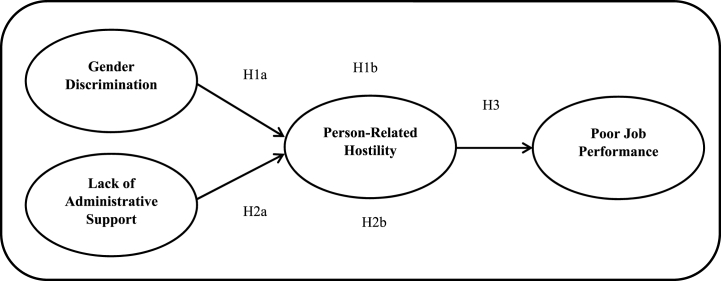
Conceptual framework.

The conceptual framework based on earlier discussed literature has been proposed in Fig. 1 to highlight the possible antecedents and outcomes of person-related hostility.

## 3 Method

### 3.1 Design

The current study is descriptive, cross-sectional, and questionnaire-based. Philosophical grounds for quantitative research are provided by post-positivism. Cresswell [69] identified this worldview as scientific technique, postpositivist/positivist research, post-positivism, and empirical science.

### 3.2 Participants and procedure

This study was conducted in fourteen large public sector hospitals from Islamabad, Lahore, Peshawar and Faisalabad. The number of patients in these public sector hospitals has been increasing over the years as the middle, and lower classes cannot afford expensive private hospitals. These hospitals provide medical care to patients in all disciplines. These hospitals have been facing a dire nursing staff shortage despite the increased patient care burden. Nurses working at different designations, such as head nurses, charge nurses and junior nurses, were included in this study. These nurses had been providing health care to patients in different departments of the hospitals. Only the chief nursing superintendents were excluded, as they manage the nursing workforce.

Questionnaires were administered to the female nurses working in public sector hospitals in Pakistan. The convenience sampling technique was used in the study as it was challenging to find a sampling frame. Sample demographics have been presented in Table 1. A total of 800 questionnaires were distributed, out of which 707 were considered for final analysis. For data analysis, the general criterion for the sample size of 300 is good, and 500 is very good [70,71]. The goodness of the sample size was also confirmed with G*Power 3.1 software, which calculated the sample size of 569 with 99% power, multiple correlations (R) 0.40 at the significance level of 0.05.

**Table 1:** Sample demographics.

Characteristics	N (Percentage)
Designation	
Head Nurse	65 (9.2)
Charge Nurse	534 (75)
Junior Nurse	108 (15.3)
Marital Status	
Married	480 (67.9)
Single	227 (32.1)
Qualification	
Matric Nursing Diploma	31 (4.4)
Inter Nursing Diploma	114 (16.1)
BSc Nursing	460 (65.1)
MSc Nursing	38 (5.4)
Any Other	64 (9.1)
Age	
20–30 years	355 (50.2)
31–40 years	266 (37.6)
41–50 years	57 (8.1)
51–60 years	29 (4.1)
Institute	
PIMS	60 (8.5)
Poly Clinic Hospital	61 (8.6)
NIRM	16 (2.5)
SIMS	69 (9.8)
Lahore General Hospital	60 (8.5)
Jinnah Hospital	60 (8.5)
Sheikh Zayed Hospital	61 (8.6)
Mayo Hospital	61 (8.6)
Lady Willington Hospital	30 (4.2)
Allied Hospital	45 (6.2)
DHQ Hospital	54 (7.6)
Lady Reading Hospital	60 (8.5)
Peshawar General Hospital	30 (4.2)
Khyber Teaching Hospital	40 (5.7)

Before data collection, participants were briefed on the aim, scope of the study, confidentiality of data, and their right to decline participation at any stage. A cover letter was attached to the questionnaire to inform respondents about the purpose of the research. It took six months to collect data from all participants, and data were collected between August 2019 and January 2020. The Negative Acts Questionnaire-Revised (NAQ-R) was adapted to measure person-related hostility. SPSS 20 and Smart PLS 3 software were used for data analysis. Through this method, complex relationships between several variables can be drawn. This software can model multiple independents and dependents.

### 3.3 Measures

#### 3.3.1 Gender discrimination

The inequality or discrimination towards a character or group of individuals because of gender identity or sex refers to gender discrimination [72]. Gender discrimination is measured by 6 items scale [73]. The sample item of gender discrimination is, i.e., *I receive fewer promotional opportunities as compared to males.*

#### 3.3.2 Lack of administrative support

Lack of administrative support or a toxic work environment is characterised by poor people management practices with profit orientation rather than people orientation. A 6 items scale developed [73] has been used to measure the lack of administrative support. The sample item for lack of administrative support is, i.e., *employees are not appreciated when they have done a good job*.

#### 3.3.3 Person-related hostility

In the present study, we measured person-related hostility as the dimension of horizontal hostility. Behavioural tendencies of person-related bullying include gossip, yelling, spreading rumors, insulting, public humiliation, intruding on one’s privacy, or ignoring someone [74]. Person-related hostility is measured through the 19 items scale adapted from the Negative Acts Questionnaire-Revised (NAQ-R) developed by Ref. [75]. The sample item for person-related hostility is, i.e., *my colleagues reveal or discuss my personal or private life in a negative way.*

#### 3.3.4 Poor job performance

Poor job performance is another outcome of horizontal hostility in the healthcare sector. Poor job performance refers to an employee’s behaviour or performance below requisite standards. Poor job performance has been measured by 3 items scale of Stamps [76]. The sample item for poor job performance is, i.e., *I am unable to deliver well-prepared or careful nursing service to the patients*.

## 4 Results

### 4.1 Correlations

Pearson correlation coefficient values were positive and significant for all variables (Table 4). Results showed that gender discrimination (r = 0.581, p < 0.05) and lack of administrative support (r = 0.496, p < 0.05) are positively associated with person-related hostility at a moderate level. The association of person-related hostility with poor job performance (r = 0.712, p < 0.05) is high. The correlation results support the hypothesis that gender discrimination and lack of administrative support are positively associated with the dependent variable, person-related hostility. Moreover, the hypothesis about the significant positive association of person-related hostility with poor job performance is also supported.

### 4.2 Reliability and validity

#### 4.2.1 Outer loading

At first, the outer loading of each item was observed (Table 2). The range of outer loading was 0.484–0.931. In the model tested, outer loadings for all items were greater than 0.5 except for one item related to lack of administrative support. Outer loading of item 11, ‘*There is no space for employees of different races and religions,*’ was 0.484, hence deleted for final analysis. For person-related hostility, item 16, ‘*My colleagues negatively comment on my work and personality to upset me,* had the highest outer loading of 0.824. Items 16, 19, 21, 24, and 29 had outer loadings above 0.80. For gender discrimination, item 3, ‘*I receive fewer promotional opportunities as compared to males’* had the highest outer loading of 0.856. Outer loadings of items 4 and 6 were also above 0.80. For poor job performance, the outer loading of item 34, *‘I am unable to deliver well-prepared or careful nursing service to the patients,* was 0.931.

**Table 2:** Outer loadings.

No	Items	Outer Loading
	**Gender Discrimination**	
1	I have to work harder to achieve tasks as compared to males.	0.734
2	At my workplace, males are more authoritative as compared to females.	0.795
3	I receive fewer promotional opportunities as compared to males.	0.856
4	My promotion is delayed due to discrimination based on gender.	0.844
5	Males use their social networks to get benefits early.	0.784
6	I feel that males get early promotions in the workplace.	0.825
	**Lack of Administrative Support**	
7	I cannot trust the management of the hospital.	0.763
8	Management withholds important information from the employees.	0.775
9	Employees are not appreciated when they have done a good job.	0.768
10	Work is not distributed fairly among all employees.	0.748
11	There is no space for employees of different races and religions.	0.484
12	Conflicts are not resolved in a fair way by management.	0.776
	**Person-related Hostility**	
	***Gossips:***	
13	My colleagues spread gossips and rumors about me.	0.676
14	My colleagues fail to respect my privacy.	0.744
15	My colleagues reveal or discuss my personal or private life in negative way.	0.799
	***Backbiting***	
16	My colleagues make insulting or offensive remarks about my person, attitudes, and private life in my absence.	0.801
17	My colleagues harm my dignity and repute and humiliate me through backbiting.	0.783
	***Negative Comments***	
18	I experience persistent criticism of my work or effort.	0.753
19	My colleagues negatively comment my work and personality to upset me	0.824
	***Telling False Stories***	
20	My colleagues make false stories about me to create misunderstandings to undermine my success	0.790
21	My colleagues indirectly attack me by telling false stories to distort my personality and work	0.822
	***Teasing and Avoiding***	
22	I am being ignored or excluded or isolated from others	0.765
23	My colleagues hint I should quit my job.	0.526
24	I am being ignored or facing a hostile reaction when I approach.	0.800
25	I am being subject of excessive teasing and sarcasm.	0.680
26	Practical jokes carried out by people you don’t get along with	0.509
	***Verbal Abuse***	
27	I experience shouting or being the target of spontaneous anger from my colleagues	0.625
28	I am humiliated or ridiculed in connection with my work	0.797
	***Non-verbal Negative Gestures***	
29	My colleagues make derogatory faces in response to a question	0.809
30	I experience eye rolling from my colleagues in response to a question	0.777
31	My colleagues express their aggression by walking with heavy feet	0.763
	**Poor Job Performance**	
32	I am unable to deliver well-prepared or careful nursing service to the patients.	0.931
33	I am unable to manage nursing activities in time.	0.912
34	I am unable to endorse and follow clinical rules, procedures and hospital policies for patient care.	0.929

#### 4.2.2 Internal consistency

For assessment of the reliability of the instrument, internal consistency was calculated through Cronbach’s alpha and Composite Reliability (Table 3). For all latent variables, Cronbach’s alpha values were calculated and ranged between 0.817 and 0.954. The values of Cronbach’s alpha above 0.7 show high internal consistency of latent variables [77]. Composite Reliability (CR) values were also calculated for all latent variables and ranged between 0.868 and 0.959. A composite reliability value higher than 0.70 is recommended by Ref. [78]. In the present study, all latent variables showed high composite reliability (CR). These findings indicate that the instruments used to measure all latent variables (gender discrimination, lack of administrative support, person-related hostility and poor job performance) are reliable as their values are within the acceptable range.

**Table 3:** Construct reliability and validity.

Construct	Cronbach Alpha	Composite Reliability (CR)	Average Variance Extracted (AVE)
Gender Discrimination	0.893	0.918	0.651
Lack of Administrative Support	0.817	0.868	0.528
Person-Related Hostility	0.954	0.959	0.555
Poor job Performance	0.914	0.946	0.854

#### 4.2.3 Convergent validity

To measure convergent validity, we calculated the Average Variance Extracted (AVE) for all latent variables (Table 3). The AVE values for all latent variables were greater than 0.5 and ranged between 0.528 and 0.854. Hair et al. [79] recommended a value of AVE greater than 0.5, showing high convergent validity for all latent variables. These findings indicate that all the instruments used to measure latent variables (gender discrimination, lack of administrative support, person-related hostility and poor job performance) are valid and that their items converge to represent the underlying construct.

#### 4.2.4 Discriminant validity

Discriminant validity is calculated through Fornell- Lacker criterion. Its value is calculated by contrasting the square root of average extracted variance (AVE) values with correlations among latent variables. Findings showed that correlation values among the latent variables were less than the square root of AVE values. The results of discriminant validity are presented in Table 4. These findings showed that all the instruments used to measure latent variables (gender discrimination, lack of administrative support, person-related hostility and poor job performance) that are not supposed to be related are unrelated.

**Table 4:** Discriminant validity (Fornell- Larker criterion).

Construct	GD	LAS	PRH	PJP
Gender Discrimination (GD)	**0.807**			
Lack of Administrative Support (LAS)	0.667	**0.727**		
Person-Related Hostility (PRH)	0.581	0.496	**0.745**	
Poor Job Performance (PJP)	0.401	0.331	0.712	**0.924**

### 4.3 Model fit

All the hypotheses were accepted, indicating significant direct relationships between exogenous (gender discrimination and lack of administrative support) and endogenous (person-related hostility and intention to leave). In the second stage, hypotheses regarding the mediating role of person-related hostility between exogenous and endogenous variables were tested.

#### 4.3.1 Evaluation of collinearity

The variance inflation factor (VIF) was calculated for all items of each variable separately to check collinearity. No collinearity issue was found as the values of VIF were less than 5, as recommended by Ref. [79]. These findings show that the independent variables (gender discrimination and lack of administrative support) are not correlated and express a linear relationship in the regression model and independently predict the value of dependent variables (person-related hostility and poor job performance).

#### 4.3.2 Path coefficients of structural model

Fig. 1 represents the structural model for the present study. Hypotheses were tested, and the significance of relationships between exogenous and endogenous variables were determined through β values. The β values were measured for each path in hypothesised models. High β values indicate a more significant impact of the exogenous latent construct on the endogenous latent construct. The T-statistics test values were also calculated to validate the value of β for its significance level. Table 5 shows the structural model’s path coefficients, including β, T-Statistic, and P-value values. For direct paths, we found a significant positive relationship between gender discrimination and person-related hostility (β = 0.451, p < 0.000), lack of administrative support and person-related hostility (β = 0.196, p < 0.000), and person-related hostility and poor job performance (β = 0.712, p < 0.000). For the measurement of indirect paths, the bootstrapping method was employed [80]. The findings revealed a significant positive mediating role of person-related hostility between gender discrimination and poor job performance (β = 0.321, p < 0.000) and between lack of administrative support and poor job performance (β = 0.139, p < 0.000).

**Table 5:** Path coefficients.

Hypotheses	Structural Path	T-Statistic	Beta Value	P-Value
H1a	Gender Discrimination -> Person-Related Hostility	10.321	0.451	0.000
H2a	Lack of Administrative Support -> Person-Related Hostility	4.151	0.196	0.000
H1b	Gender Discrimination -> Person-Related Hostility -> Poor Job Performance	10.197	0.321	0.000
H2b	Lack of Administrative Support -> Person-Related Hostility -> Poor Job Performance	4.099	0.139	0.000
H3	Person-Related hostility -> Poor Job Performance	30.514	0.712	0.000

#### 4.3.3 Coefficient of determination R^2^ and f^2^ effect size

The coefficient of determination R^2^ determines the overall effect size and variance explained by the endogenous construct. Hence, the model’s predictive accuracy is measured through the coefficient of determination R^2^. The value of the coefficient of determination for endogenous latent variables was calculated. The value of R^2^ was considered moderate (0.359) for person-related hostility and poor job performance (0.509) according to the criteria provided by Refs. [79,81]. The f^2^ determines the change in the value of R^2^ for the inclusion or exclusion of construct in the model. When the exogenous latent construct is excluded from the model, its significant impact on the value of the endogenous latent construct is described through the f^2^ effect size. For exogenous latent variables, the value of the f^2^ effect size was calculated. Hence, the f2 effect size value is (f^2^ = 0.176) for gender discrimination and (f^2^ = 1.030) for person-related hostility, which strongly impacts endogenous latent variables. For lack of administrative support, its value is 0.033 showing a low impact on the endogenous latent variable.

#### 4.3.4 Evaluation of predictive relevance Q^2^

Predictive relevance Q^2^ measures prediction error. The value of prediction error higher than zero shows the predictive relevance of the model with specific variables. The Q^2^ values were determined for person-related hostility (Q^2^ = 0.187) and poor job performance (Q^2^ = 0.231). Q^2^ values indicate higher predictive relevance for the selected endogenous latent variables because of exogenous variables.

## 5 Discussion

This study proved to be in line with Freire’s oppression paradigm [26,27], which stated that oppression leads to violence, which in turn leads to oppressed violence. When the oppressed gain equal power and position, they start oppressing, and then the oppressors feel oppressed. They become self-centred and do not treat everyone fairly. Thus, Freire’s theory of oppression has been used as an underpinned theory for developing the conceptual framework. The findings revealed that gender discrimination and lack of administrative support are the organisational antecedents of person-related hostility and further contribute to poor job performance. The current study has highlighted that nurse managers and hospital administrators are responsible for creating an atmosphere conducive to professional communication and collaboration among healthcare workers to improve patient care quality and job performance. Organisations have to face many internal and external challenges parallelly.

In the present study, data were collected from head nurses, charge nurses and junior nurses working in public sector hospitals situated in four main cities of Pakistan. H1a and H1b were hypothesised to check the impact of gender discrimination on person-related hostility and the mediating effect of person-related hostility on the relationship between gender discrimination and poor job performance; the results showed that the proposed hypotheses are true. There are numerous unfortunate consequences of discriminatory practices, including helplessness, psychological stress, and frustration. These adverse feelings result in negative work behaviours and inhibit individuals from career development and professional goal achievement, and current results are aligned with earlier literature [42,43]. In the health sector, nurses experience moral and psychological distress as they feel incompetent or their colleagues consider their nursing practices objectionable. Nurses refer to the psychological group that traditionally experiences discrimination and fights against it to attain fair treatment and social justice. Therefore, this study has been conducted in a contextual setting of the nursing profession. Discrimination in the workplace is viewed as aggressive behaviour, such as any unprofessional behaviour, conflict, or hostility extending from verbal abuse to physical abuse, which results in enhanced violent behaviour horizontally [36]. Such negative behaviours adversely affect inter-professional collaboration, communication, and teamwork [32]. Given that the consequence of contentious conduct between physicians, nurses, and other healthcare employees averts collaboration, discussion, teamwork, and interchange of information, it may impact patient care and dynamics such as medical mistakes, safety risks, happening of hostile events, and poor healthcare quality which ultimately affect job performance [42].

H2a and H2b were hypothesised to check the impact of lack of administrative support on person-related hostility and the mediating effect of person-related hostility among the relationship between lack of administrative support and poor job performance; the results showed that the proposed hypotheses were true. The study confirmed that the feelings of uncertainty due to lack of administrative support provoke subordinates to inflict horizontal hostility to express themselves more powerfully [50,51]. Results also proved that lack of administrative support is a significant reason for dejection among the oppressed [46,47]. The oppressed people seem emotionally helpless, and earlier, they realise their reliance, expressing their frustration and desperation at times by drinking at home, which may be the only way of exit for them [48,49]. Two environments are incompatible and result in conflicts and dissatisfaction; the environment where a nurse is trained and the environment where they professionally work after getting the license. In the nursing literature, the disagreement between predetermined concepts and actual world practice is known as transition or reality shock. Moreover, new graduate nurses look for employment opportunities and situations that help and support their persistent knowledge through internships, orientation programs, education, and laddering job positions that develop their competence and confidence and improve their clinical expertise. As continuing education and up-to-date skills are prerequisites for professional development, new graduate nurses may expect administrative support to enhance their competencies throughout their careers.

H3 was hypothesised to check the direct effect of person-related hostility on poor job performance; results showed that the proposed hypothesis is true. Results also highlighted that female recipients of hostility try to avoid interfacing with colleagues who target them through abuse and insulting behaviours [58,59]. This hostile behaviour is not confined to the deteriorated relationship between the two groups. It also affects individuals who are witnesses, and teamwork is sabotaged in the form of poor job performance [60,61].

## 6 Conclusion

This study provides empirical evidence for a significant and positive relationship between gender discrimination and lack of administrative support with person-related hostility and poor quality of patient care. This study highlights the importance of hospital administrators and nurse managers who are responsible for providing a peaceful work environment and preventing incivility and violence in healthcare settings. An organisational culture with administrative support and gender equality will increase positive affectivity, job satisfaction, and commitment among nursing professionals. Teamwork can be a positive factor in enhancing organisational efficiency and the probability that the group will control the individual through aggressive behaviour. This will improve the risk of revolving some individuals into scapegoats and endangering them to hostility. In contrast, leaders can develop a healthy work environment for employees by promoting a collaborative environment based on mutual trust and respect. Empowerment and training can result in a positive learning environment. The employees must be educated about appropriate professional behaviour with the prominence of respect as defined by the code of conduct.

### 6.1 Theoretical implications

This study is the maiden study that highlights the issue of person-related hostility and empirically tests a conceptual framework for the understanding of its antecedents and outcomes. This research study contributes to the literature in two ways. First, it has highlighted the significance of gender discrimination and lack of administrative support as the primary organisational-level antecedents of person-related hostility. It provides empirical evidence for the relationship of variables and can be considered a significant theoretical contribution to the literature of Freire’s oppression theory [26,27]. Second, applying the research model explains the outcome of person-related hostility, leading to poor job performance. This study will help academicians to understand person-related hostility, its behavioural tendencies, and its role in job dissatisfaction and demotivation of female nursing professionals. The questionnaire has been adapted to measure person-related hostility in Pakistan. This questionnaire has been validated in the context of Pakistan as the measures of reliability and validity are in the acceptable range. This questionnaire can also be used to collect data in other South Asian countries.

### 6.2 Practical implication

This study has significant practical implications for nurse managers, hospital administrators, and policymakers. Hostility among nursing professionals can be addressed by preventing a culture of discrimination and injustice in the healthcare setting. This research provides baseline information for nurse managers and administrators to understand behavioural tendencies and the organisational factors contributing to person-related hostility. These hostile behaviours affect the psychological health and social well-being of employees. Therefore, it will help to make interventions through policies and procedures for its prevention. Gender discrimination and lack of administrative support have been highlighted as the main antecedents of violence and incivility among female nurses. A culture of collaboration and cooperation in healthcare may be developed by preventing these factors. To enhance the quality of patient care, nurse managers and hospital administrators are responsible for establishing and providing a peaceful work environment that may result in professional communication and collaboration among healthcare employees. HR strategies for nursing professionals should be revised with more focus on timely promotions, pay increases, other benefits, rewards, recognition, and career progression opportunities.

### 6.3 Limitations and future research directions

This research study was conducted in public sector hospitals in the main cities of Pakistan. Firstly, in small cities and towns, gender inequality seems higher because of low education levels and social and cultural norms. Therefore, the intensity of hostile behaviours among female nurses may be higher compared to large cities. Secondly, data was collected from four main cities, including Islamabad, Lahore, Faisalabad and Peshawar. It shows representation from Capital Territory, Punjab and Khyber Pukhtunkhawa provinces. Future research may study person-related hostility among female nurses working in hospitals in semi-urban areas and small towns with low literacy rates. Some dependent variables like burnout, mental well-being, and mental health can also be studied for practical and theoretical enhancement and replication of the study. This phenomenon may also be studied in other female professions, including teachers, doctors, etc., for a comprehensive policy decision to control the possible effect of gender discrimination and lack of administrative support.

### 6.4 Policy recommendations

At the Government level, anti-harassment laws should be established to reduce hostility in the workplace. The strategies regarding gender discrimination and administrative support to females should be devised to improve workplace conditions for nursing professionals and include a zero-tolerance policy for the prevalence of hostility. Staff should be empowered to exclaim without fear of retaliation. There is a need to devise a satisfactory job structure for nurses, including better pay scales, training and promotional opportunities, study leaves for higher qualifications and in-time promotions by the health department. To meet the demand of increasing patients in public sector hospitals, the recruitment of nurses should be ascertained to overcome the shortage. A precise mechanism must be developed for time training and promotions.

## Author contribution statement

Nadia Noor, PhD: Conceived and designed the experiments; Performed the experiments; Wrote the paper.

Saqib Rehman, PhD: Analyzed and interpreted the data; Wrote the paper.

Yasmeen Ahmed, PhD; Muhammad Sarmad, PhD; Rashid Mehmood, PhD: Contributed reagents, materials, analysis tools or data; Wrote the paper.

## Funding statement

This research did not receive any specific grant from funding agencies in the public, commercial, or not-for-profit sectors.

## Data availability statement

Data will be made available on request.

## Declaration of interest’s statement

The authors declare that they have no known competing financial interests or personal relationships that could have appeared to influence the work reported in this paper.
