# Supplementary material for: Discriminatory practices and poor job performance: A study of person-related hostility among nursing staff
Source: Heliyon. 2023 Mar 7;9(3):e14351. doi: 10.1016/j.heliyon.2023.e14351 (PMC10020078; doi:10.1016/j.heliyon.2023.e14351)
Supplement: Multimedia component 1 [file mmc1.docx]

**Discriminatory Practices and Poor Job Performance: A Study of Person-Related Hostility Among Nursing Staff**

**Note:** Please mention your level of agreement on the following statements on five-point Likert scale (1 = strongly disagree; 2 = disagree; 3 = neutral; 4 = agree; 5 = strongly agree).

|  | **Gender Discrimination** | | | | | |
| --- | --- | --- | --- | --- | --- | --- |
| 1 | I have to work harder to achieve tasks as compared to males. | **1** | **2** | **3** | **4** | **5** |
| 2 | At my workplace, males are more authoritative as compared to females. | **1** | **2** | **3** | **4** | **5** |
| 3 | I receive less promotional opportunities as compared to males. | **1** | **2** | **3** | **4** | **5** |
| 4 | My promotion is delayed due to discrimination based on gender. | **1** | **2** | **3** | **4** | **5** |
| 5 | Males use their social network to get benefits early. | **1** | **2** | **3** | **4** | **5** |
| 6 | I feel that males get early promotions at workplace. | **1** | **2** | **3** | **4** | **5** |
|  | **Lack of Administrative Support** | | | | | |
| 7 | I cannot trust the management of the hospital. | **1** | **2** | **3** | **4** | **5** |
| 8 | Management withholds important information from the employees. | **1** | **2** | **3** | **4** | **5** |
| 9 | Employees are not appreciated when they have done a good job. | **1** | **2** | **3** | **4** | **5** |
| 10 | Work is not distributed fairly among all employees. | **1** | **2** | **3** | **4** | **5** |
| 11 | There is no space for employees of a different race and religion. | **1** | **2** | **3** | **4** | **5** |
| 12 | Conflicts are not resolved in a fair way by management. | **1** | **2** | **3** | **4** | **5** |
|  | **Person Related Hostility** | | | | | |
|  | **Gossips:** |  |  |  |  |  |
| 13 | My colleagues spread gossips and rumors about me. | **1** | **2** | **3** | **4** | **5** |
| 14 | My colleagues fail to respect my privacy. | **1** | **2** | **3** | **4** | **5** |
| 15 | My colleagues reveal or discuss my personal or private life in negative way. | **1** | **2** | **3** | **4** | **5** |
|  | **Backbiting** |  |  |  |  |  |
| 16 | My colleagues make insulting or offensive remarks about my person, attitudes, and private life in my absence. | **1** | **2** | **3** | **4** | **5** |
| 17 | My colleagues harm my dignity and repute and humiliate me through backbiting. | **1** | **2** | **3** | **4** | **5** |
|  | **Negative Comments** |  |  |  |  |  |
| 18 | I experience persistent criticism of my work or effort. | **1** | **2** | **3** | **4** | **5** |
| 19 | My colleagues negatively comment my work and personality to upset me | **1** | **2** | **3** | **4** | **5** |
|  | **Telling False Stories** |  |  |  |  |  |
| 20 | My colleagues make false stories about me to create misunderstandings to undermine my success | **1** | **2** | **3** | **4** | **5** |
| 21 | My colleagues indirectly attack me by telling false stories to distort my personality and work | **1** | **2** | **3** | **4** | **5** |
|  | **Teasing and Avoiding** |  |  |  |  |  |
| 22 | I am being ignored or excluded or isolated from others | **1** | **2** | **3** | **4** | **5** |
| 23 | My colleagues hint I should quit my job. | **1** | **2** | **3** | **4** | **5** |
| 24 | I am being ignored or facing a hostile reaction when I approach. | **1** | **2** | **3** | **4** | **5** |
| 25 | I am being subject of excessive teasing and sarcasm. | **1** | **2** | **3** | **4** | **5** |
| 26 | Practical jokes carried out by people you don't get along with | **1** | **2** | **3** | **4** | **5** |
|  | **Verbal Abuse** |  |  |  |  |  |
| 27 | I experience shouting or being the target of spontaneous anger from my colleagues | **1** | **2** | **3** | **4** | **5** |
| 28 | I am humiliated or ridiculed in connection with my work | **1** | **2** | **3** | **4** | **5** |
|  | **Non-verbal Negative Gestures** |  |  |  |  |  |
| 29 | My colleagues make derogatory faces in response to a question | **1** | **2** | **3** | **4** | **5** |
| 30 | I experience eye rolling from my colleagues in response to a question | **1** | **2** | **3** | **4** | **5** |
| 31 | My colleagues express their aggression by walking with heavy feet | **1** | **2** | **3** | **4** | **5** |
| **Poor Job Performance** | | | | | | |
| 32 | I am unable to deliver well-prepared or careful nursing service to the patients. | **1** | **2** | **3** | **4** | **5** |
| 33 | I am unable to manage nursing activities in time. | **1** | **2** | **3** | **4** | **5** |
| 34 | I am unable to endorse and follow clinical rules, procedures and hospital policies for patient care. | **1** | **2** | **3** | **4** | **5** |
